# The Impact of the Introduction of the Breast Unit Model in a Northwestern Italian Region

**DOI:** 10.3390/healthcare10081512

**Published:** 2022-08-11

**Authors:** Laura Paleari, Federico Tassinari, Matteo Astengo, Daniela Amicizia, Chiara Paganino, Gabriella Paoli, Paolo Pronzato, Filippo Ansaldi

**Affiliations:** 1 Research, Innovation, HTA Unit, Liguria Health Authority (A.Li.Sa.), Genoa 16121, Italy; 2 Health Planning Unit, Liguria Health Authority (A.Li.Sa.), Genoa 16121, Italy; 3 Department of Health Sciences, University of Genoa, Genoa 16121, Italy; 4 Health Planning Unit, Ospedale Policlinico San Martino—IRCCS, Genoa 16121, Italy; 5 Department of Medical Oncology, UO Oncologia Medica 2, IRCCS AOU San Martino-IST, Genoa 16121, Italy

**Keywords:** breast cancer, outcome indicators, organizational model

## Abstract

Breast cancer is the most common tumor in middle-aged and older women. In 2003, the European Parliament recommended to Member States that all women with breast cancer should be treated by a multidisciplinary team and that a network of certified breast centers be organized (the centers have been called Breast Units (BUs)). With the present study, we aim to explore the impact of the introduction of the BU organizational model in the Liguria region, Italy, through different outcome indicators. An explorative retrospective analysis was conducted through the period from 2013 to 2019 to assess the impact of the introduction of the BU model in our region. We identified two periods: before (2014–2015) and after (2017–2018) the introduction of this organizational model to assess its value impact through the definition of six measurable outcome indicators. Length of hospitalization, repeated specialist outpatient diagnostic procedures and the rate of subjects who started radiotherapy treatment within 60 days improved after the introduction of BUs. The passive health migration rate only improved significantly for one local health unit (LHU), while reintervention and diagnosis–surgery time did not show any enhancement after the introduction of the BU model. The BU model seems to provide an increase in several aspects of the healthcare offered to breast cancer patients in Liguria, specifically in those areas where a shared guideline could assist healthcare workers. Future research, such as pilot studies, are needed to assess the impact of the introduction of the BU model in our reality.

## 1. Introduction

**Figure 1:**
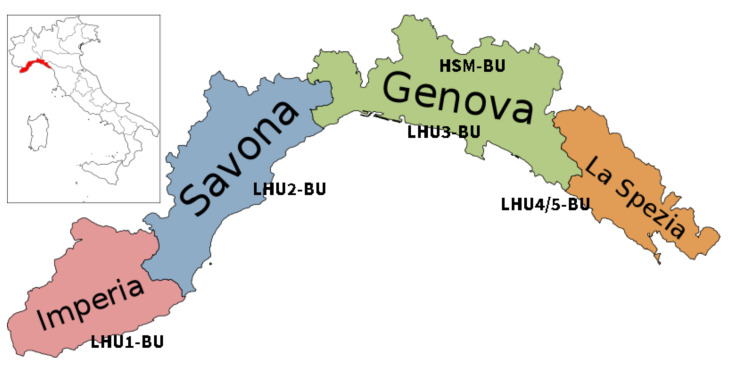
Map of the geographical area of the study site with the localization of the five Ligurian Breast Units. LHU1-BU: Breast Unit of Local Health Unit 1; LHU2-BU: Breast Unit of Local Health Unit 2; LHU3-BU: Breast Unit of Local Health Unit 3; HSM-BU: Breast Unit of IRCCS San Martino Policlinic Hospital; LHU4/5-BU: Breast Unit of Local Health Unit 4 and 5.

Breast cancer is the most common tumor in middle-aged and older women [1,2,3]. Worldwide in 2018, it is estimated that there were 2,088,800 (95% CI: 2,003,700–2,177,600) new cases of breast cancer in the female population and an age-standardized incidence rate of 46.3 per 100,000, with an age-standardized mortality rate of 13.0. In Europe, the age-standardized incidence rate varied from 92.6 per 100,000 in western Europe to 80.3 per 100,000 in southern Europe [1,2,3]. In Italy, an age-standardized breast cancer incidence rate of 92.8 per 100,000 has been estimated [1,2,3], consistent with the data that show breast cancer incidence is highest in high economic European countries, including most of northern and western Europe, along with Italy and Malta from southern Europe [4]. Starting from the 1990s, it has been observed that breast cancer mortality in Europe has experienced a declining trend [5]. This reduction has been greater in the northern than in the eastern regions, where lower mortality rates were observed in the past [5]. The reasons for such a positive trend are principally related to diagnostic and therapeutic improvements [6]. Currently, the pattern observed in the past has been inverted, with rates of breast cancer mortality being higher in central and eastern countries, where they were lower in the past [5]. This could be explained by the political, economic and social changes that have occurred in these countries over the last three decades, and their impact on health policy [7]. In 2003, the European Parliament [8,9] recommended to Member States that a multidisciplinary team should treat all women with breast cancer and that a network of certified breast centers be organized. In 2006, a new resolution [10] established that these centers had to be created by 2016. A call-to-action statement was published for an organizational model that could increase the survival rate and quality of life of women with breast cancer [11]. In 2014, Italy implemented this resolution [12], and 2 years later, Liguria was one of the first Italian regions to introduce the Breast Unit (BU) model (Figure 1) [13]. Specifically, a BU is a multidisciplinary breast care center specializing in the management of women with breast problems, including breast cancer. In a broader sense, this is a care path that goes from screening activities to diagnostic investigations, from surgery to the definition of the therapeutic strategy, from psychophysical rehabilitation to long-term controls (follow-up), and up to genetic counseling. The creation of a multidisciplinary breast center is planned for every 250,000 inhabitants; each center must treat at least 150 new cases each year and must have at least a team of six dedicated professionals (i.e., radiologist, surgeon, pathologist, oncologist, radiotherapist, data manager) [7,8,9]. High-quality services are essential to optimize the treatment results for women with breast cancer. In fact, it has been shown that accurate training, specialization, high surgical case history and a multidisciplinary approach, involving many different specialists, nursing staff and supporting staff members [14], can achieve this goal. It is of primary importance to develop structured models of care in order to avoid disordered and inconsistent patient management and to improve adherence to evidence-based guidelines. Recently, a report was published of a European survey on the implementation of BUs in accordance with the 2006 European Guidelines that defined the BU requirements [15]. The results of this survey underlined some variation across Europe with regard to the number of requirements implemented. The two most commonly implemented requirements across all countries were the multidisciplinary case management (MDM) meetings and the provision of appropriate adjuvant therapy, present in 19 out of 25 countries (76%) [15]. Thus, the enhancements in health consequences for women whose BC has been debated in MDM meetings have been evaluated. The results show that patients evaluated in MDM meetings are more likely to be treated with standard best practices, even if there is very low evidence that MDM compared to non-MDM reduces 5-year mortality from BC. Moreover, the establishment of extra psychological support, physiotherapy for treatment of lymphedema, radiotherapy equipment, management of benign disease, training requirements for core teams, communication of diagnosis and prosthesis were implemented in more than half of the reporting countries. Interestingly, the training for the core team requisite had been implemented in only 13 countries, although it is a mandatory requirement for BUs in accordance with the 2006 European Guidelines. Finally, the five requirements less frequently implemented were: the management of advanced and recurrent BC, core team, continuing medication, new patient clinics, and the volume requirement—the last three of which are considered obligatory by the 2006 European Guidelines [15]. In line with this, the European Society of Mastology (EUSOMA) is committed to fostering BUs in Europe, according to the standards set out in its document “The requirements of a Specialist Breast Centre” [16]. In order to make sure that BUs follow these requirements and increase the standards of care, EUSOMA promotes the voluntary Certification Process by BUs Certification [16]. To date, many European BUs are neither certified nor meet EUSOMA standards. Interestingly, in 2010, the German Cancer Society developed a volunteer certification initiative for the European cancer centers in order to improve the quality of care within certified networks [17]. The program, starting in Germany, spread to all EU member states and by March 2020, a total of 1462 German cancer centers and 104 other cancer centers in Europe and internationally had been certified and joined the program [17,18]. To date, our BUs are all certified for quality, and since the adherence to recognized guidelines and the constant monitoring of performance throughout properly designed indicators are strategic tools to improve the quality of care, here we propose a set of six indicators to evaluate the impact of BU introduction in our region. In fact, as in clinical practice, clinical trials are performed to evaluate, for example, the advantages of introducing a new treatment, so new planning strategies should be assessed to apply the concept of evidence-based medicine (EBM) to the field of health planning and management. In recent years, several indicators have been proposed to explore the quality of care, dividing them into three principal categories: setting, process and outcome [19,20,21].

With the present study, we aim to assess the impact of the introduction of the BU organizational model in the Liguria region through different outcome indicators designed ad hoc on the basis of the regional decree *n* 622/2016 and the National Outcomes Programme (NOP) [22,23]. The indicators were: reintervention within 90 days of breast cancer surgery; passive health migration rate; time elapsed between a first diagnostic examination, when present, and surgery; length of hospitalization; repeated specialist outpatient diagnostic procedures relating to breast cancer; and the number of subjects who started radiotherapy treatment within 60 days of surgery in the absence of chemotherapeutic treatment.

## 2. Materials and Methods

Liguria, the oldest Italian region, has 1.532.980 inhabitants with an aging index of 257.3%. Healthcare is organized into five LHUs numbered from 1 to 5 from west to east, with LHU 3 being the territory of the capital city of the region [24]. An explorative retrospective analysis was conducted through the period from 1 December 2013 to 30 June 2019, identifying two periods as before (2014–2015) and after (2017–2018) the introduction of the BU model in the Liguria region.

We used the following indicators to compare the two reference periods:

- Reintervention within 90 days of breast cancer surgery: calculated as the number of secondary surgeries occurring within 90 days of a primary surgery divided by the number of primary surgeries among residents operated on in Liguria;
- Passive health migration rate: the number of surgeries performed on Liguria residents in another Italian region over the number of surgeries on Liguria residents;
- Diagnostic–therapeutic time: the days elapsed between the first diagnostic intervention and the day of the surgery;
- Length of hospitalization: the days elapsed between hospitalization and discharge;
- Repeated specialist outpatient diagnostic procedures: we only considered closed percutaneous needle biopsy of the breast; mammography; magnetic resonance imaging; breast histo-cytopathological examination (stereotaxic biopsy or lumpectomy); and general physical examination (surgical or oncological branch). Each procedure occurring beyond the first six months preceding the surgery was considered repeated. The unitary cost for each procedure was deducted from the unique regional tariff;
- Number of subjects who started radiotherapy treatment within 60 days of surgery in the absence of chemotherapeutic treatment: number of subjects who underwent radiotherapy treatment (ICD-9CM 89.7 C1/89.01 P 70/89.03) within 60 days of surgery in the absence of chemotherapy.

The data were obtained through the administrative healthcare “data warehouse” regional service of the Hospital Discharge Records (HDRs) and the flow of outpatient visits. All patients were monitored from the sixth month preceding hospital admission. Subjects were included on the basis of the International Classification of Diseases 9th Revision, Clinical Modification (ICD-9-CM) diagnosis and procedure codes, selecting patients who were surgically treated with a primary or secondary diagnosis (ICD-9-CM codes 174.xx, 198.81 or 233.0) and a primary or secondary procedure (ICD-9-CM codes 85.2 *; 85.33; 85.34; 85.35; 85.36 or 85.4 *) [25]. We used the ICD-9 classification because in Italy, ICD-10 classification is not currently adopted. All analyses were performed using Epi-Info 7.2 (Centers for Disease Control and Prevention (CDC) Atlanta, GA, USA).

## 3. Results

Between 2014 and 2018, 8509 breast cancer patients in the Liguria region (3419 in 2014–2015, 1692 in 2016 and 3398 in 2017–2018) had 9078 surgeries (3615 in 2014–2015, 1874 in 2016 and 3589 in 2017–2018). The mean and median age of the patients were 64.40 (Std. 13.77) and 66 (IQR 53–75).

### 3.1. Reintervention within 90 Days of Breast Cancer Surgery

The results were stratified for the two different periods and for type of surgery. A statistical analysis was performed with χ2 test, but no statistical difference was found for the two groups, as shown in Table 1.

### 3.2. Passive Health Migration Rate

In the reference period, 1746 Liguria patients had a surgical intervention in another Italian region (73% in Lombardia) with an overall passive health migration rate of 19.2%. The decision to be treated in another region is statistically associated with a younger age (Kruskal–Wallis test, *p* << 0.01) and the place of residence inside the region: patients resident in eastern Liguria are more likely to be operated in another region (χ2 test, *p* << 0.01). While the overall passive health migration rate did not change statistically significant from 2014–2015 (19.70%) to 2017–2018 (17.94%) we found an overall a significant improvement in the suburban districts. The overall data without LH3 improved its rates from 25.18% in 2014–2015 to 20.65% in 2017–2018 (χ2 test *p* << 0.01). Table 2 presents the passive health migration rate.

### 3.3. Diagnostic–Therapeutic Time

In 2014–2015, the mean and median diagnostic–therapeutic time resulted in, respectively, 74.21 days (Std. 41.03) and 64 days (IQR 44–98), while in 2017–2018, the mean and median values were 73.29 days (Std. 37.48) and 65 days (IQR 47–92). No statistical difference between the two groups was found with the Kruskal–Wallis test.

### 3.4. Length of Hospitalization

The length of hospitalization changed significantly in the two analyzed periods: in 2014–2015, patients stayed in hospital an average of 4.26 days (Std. 9.34) and a median of 2 days (IQR 1–4), while in 2017–2018, patients had a mean length of stay of 2.89 days (Std. 6.03) and a median length of stay of 1 day (IQR 1–3). The Kruskal–Wallis test highlighted a difference between the two groups for *p* << 0.01. The Std. values in both reference periods indicate a variability in terms of the length of hospitalization among LHUs; nonetheless, after the introduction of the BUs, this inconsistency seems to be reduced.

### 3.5. Repeated Specialist Outpatient Diagnostic Procedures

In 2014–2015, breast cancer patients in the Liguria region had 2903 therapeutic diagnostic paths, and 541 of these had at least one repeated specialist diagnostic outpatient test. In 2017–2018, the number of therapeutic diagnostic pathways was 2945 and the number of paths with at least one repeated specialist diagnostic outpatient test fell to 253 (χ2 test, *p* << 0.01). Closed percutaneous needle biopsy, magnetic resonance imaging of the breast and general physical examination (surgical branch) were the types of repeated test that had the greatest decrease. The data are summarized in Table 3. This reduction led to a cost decrease for repeated examinations per single path from EUR 11.4 in 2014–2015 to EUR 4.8 in 2017–2018.

### 3.6. Rate of Subjects Who Started Radiotherapy Treatment within 60 Days of Surgery in the Absence of Chemotherapy

In 2014–2015, 175 of 1021 (17.14%) subjects started radiotherapy treatment within 60 days of surgery, while in 2017–2018, the rate was 60.21% (675 of 1121 subjects) (χ2 test, *p* << 0.01).

## 4. Discussion

Liguria is one of the first Italian regions that realized the BU model in 2016 for improving the clinical outcomes and the quality of services for breast cancer patients. In the Liguria region, the BUs are structured as follows: five surgical centers and a medical oncology/radiotherapy network are at the base of the organizational model; furthermore, within the disease management team (DMT), all further skills are ensured up to fertility preservation and oncogenetic counseling. The present explorative retrospective study aims to evaluate the impact of the introduction of this organizational model in our region through six outcome indicators chosen on the basis of the regional decree *n* 622/2016 and the NOP [22,23]. The gold standard for surgical treatment is conservative surgery and its most frequent complication is the need of reintervention. The 90-day reoperation rate for a conservative breast cancer intervention is one of the indicators proposed by the NOP [22]. Isaacs and colleagues [25] observed that reintervention rates following a first conservative surgery within 90 days ranged from 39.3% in 2003–2004 to 23.1% in 2011–2013 in New York State. Valero et al. [26] analyzed data from two centers in Boston with a high volume of breast cancer patients, and reported a reoperation rate after a conservative breast cancer surgery, for the period 2016–2017, equal to 13.2%. Reintervention rates, for conservative surgeries only, in our region were 6.60% and 7.02%, respectively, in 2014–2015 and 2017–2018, and although there has been no improvement, the data still suggest the high quality of the surgical skills. The containment of the passive health migration rate is a crucial matter for the regional healthcare economy. In fact, the surgical interventions that take place in a region on resident patients are reimbursed using the unique regional tariff, while the same type of intervention that takes place under the extra-regional regime is reimbursed with the unique national conventional tariff. This entails, for the same performance, a higher cost that the patient’s region of residence must reimburse to the provider region [27]. Our results showed a significant reduction in the passive health migration rate in the suburban area of the region from 25.18% (2014–2015) to 20.65% (2017–2018) after the introduction of the BU model. Both length of hospitalization and repeated specialist outpatient diagnostic procedures improved after the introduction of the BU model in our region as a result of a better delineated diagnostic pathway for the patients. The improvements shown in the earlier start of radiotherapy treatment, from 17.14% (2014–2015) to 60.21% (2017–2018), confirm the benefits. On the other hand, the rate of reinterventions and the diagnostic–therapeutic time did not benefit from the introduction of the BU model; one explanation is that the organizational model did not improve what may be a structural bottleneck caused by, for instance, a lack of staff and machinery (i.e., LINAC for radiotherapy). Despite the results of our study suggesting that the BU model provided an increase in several aspects of the healthcare offered to patients in our region, further improvement actions are required to increase the quality of these. In fact, in several papers, EUSOMA defined the requirements of BUs and the guidelines on the importance of training for health professionals dealing with BC [28,29,30]. In line with this, the European School of Oncology (ESO), in co-operation with Ulm University, Germany, has developed a structured course, named “Certificate of Competence in Breast Cancer (CCB)” with the aim of providing BC specialists with multidisciplinary education [31]. However, in Europe, due to heterogeneous training systems across countries, a standardized training program is absent and fellowship programs in breast surgical oncology are limited to specific countries [31]. In 2018, the major European societies answered to this requirement developing the European Breast Surgical Oncology (BRESO) project, which foresees the theoretical and practical knowledge curriculum for European Breast Surgeons [32]. The application of this program has already given results, indicating how surgeon specialization results in better oncological outcomes and increased patient satisfaction [32].

## 5. Conclusions

Healthcare planning is a largely underestimated research field. However, with the constant increase in technology and recorded data in the last decade, it could provide a useful tool for stakeholders to refine several aspects of the healthcare process.

The Hub and Spoke model, updated diagnostic–therapeutic pathways and harmonized healthcare delivery across the region could all improve the services received by patients, mainly in terms of outcomes [33].

The BU model was shown to provide enhancements in several aspects of the healthcare offered to breast cancer patients in the Liguria region, particularly in those areas where shared guidelines could assist healthcare workers. On the other hand, it was less effective in those areas where the bottleneck was caused by factors independent of the regional health organization.

## Figures and Tables

**Table 1:** Reintervention rates within 90 days of breast cancer surgery (period: before (2014–2015) and after (2017–2018) BU introduction).

	2014–2015 *N* (%)	2017–2018 *N* (%)	*p*-Value
**Any Surgery**	134/2746 (4.88%)	144/2787 (5.17%)	0.669
**Conservative Surgery**	130/1970 (6.6%)	134/1908 (7.02%)	0.645
**Invasive Surgery**	4/776 (0.52%)	10/879 (1.14%)	0.267

*N* = number of surgeries.

**Table 2:** Passive health migration rates for Local Health Unit (LHU) and period (before (2014–2015) and after (2017–2018) BU introduction).

	2014–2015 *N* (%)	2017–2018 *N* (%)	*p*-Value
**LHU 1**	81/381 (21.26%)	64/398 (16.08%)	0.1479
**LHU 2**	126/639 (19.72%)	123/695 (17.7%)	0.4752
**LHU 3**	227/1689 (13.44%)	246/1662 (14.8%)	0.351
**LHU 4**	66/347 (19.02%)	59/334 (17.66%)	0.7786
**LHU 5**	212/559 (37.92%)	152/500 (30.4%)	0.0816
**Overall**	712/3615 (19.7%)	644/3589 (17.94%)	0.1229
**Overall without LHU3**	485/1926 (25.18%)	398/1927 (20.65%)	0.009

*N* = number of patients.

**Table 3:** Repeated specialist outpatient diagnostic procedures and period (before (2014–2015) and after (2017–2018) BU introduction).

	2014–2015	2017–2018	*p*-Value
Closed percutaneous needle biopsy of the breast	417	115	
Mammography	60	81	
Magnetic resonance imaging	32	11	
Breast histo-cytopathological examination (stereotaxic biopsy or lumpectomy)	30	53	
General physical examination (surgical or oncological branch)	113	52	
Overall	652	312	
Liguria patients that had at least one repeated specialist diagnostic outpatient test	541	253	<<0.01

## Data Availability

The data were extracted from the health consultation “Data Warehouse” service that is accessible from the portal www.liguriainformasalute.it through the identification of the enabled operator.
